# Prevailing Clusters of Canine Behavioural Traits in Historical US Demand for Dog Breeds (1926–2005)

**DOI:** 10.3390/ani8110197

**Published:** 2018-11-06

**Authors:** Bethany Wilson, James Serpell, Harold Herzog, Paul McGreevy

**Affiliations:** 1 Sydney School of Veterinary Science, Faculty of Science, University of Sydney, Sydney 2006, Australia; paul.mcgreevy@sydney.edu.au; 2 School of Veterinary Medicine, University of Pennsylvania, Philadelphia, PA 19131, USA; serpell@vet.upenn.edu; 3 Department of Psychology, Western Carolina University, Cullowhee, NC 28723, USA; herzog@wcu.edu

**Keywords:** canine behaviour, canine behaviour epidemiology, pedigree dogs, canine anxiety, canine aggression, canine separation anxiety, C-BARQ

## Abstract

**Simple Summary:**

The popularity of various breeds of dog and, by extension, the ancestors of at least a portion of the crossbred population varies over time. As pedigree dogs tend to exhibit breed-specific behaviour, the loads of both adaptive and less adaptive canine behaviours within society might also be expected to vary with trends in popularity, assuming that a breed in decline is not always replaced by increasing demand for a new similarly tempered breed. Using average breed behaviours from a large online survey of dog behaviour, we organised 82 pedigree breeds into six clusters and tracked the absolute and relative numbers of American Kennel Club registrations of breeds in these behaviour clusters in 1926–2005. Our data show that there is lability in the demand for breeds of the six behavioural clusters over time. Regardless of whether behavioural differences are causative of changes in demand or merely a consequence of it, changes in the types of behaviour in the pedigree population have implications for urban planning, the demand for dog training and veterinary behavioural services and the nature of human–canine interactions. Shifts in breed demand reveal an important aspect of canine behavioural epidemiology worthy of future study.

**Abstract:**

Drawing on American Kennel Club (AKC) puppy registration numbers for approximately 82 varieties of pedigree dogs between 1926 and 2005, the current article analyses behavioural reports on 32,005 dogs of these varieties reported through the Canine Behavioural Assessment and Research Questionnaire (C-BARQ). Cluster analysis of C-BARQ scores indicates that the 82 breeds fell into six clusters. Average scores for each of the 14 behavioural subscales and 22 miscellaneous traits in C-BARQ were calculated for each cluster, and the breeds in each cluster with average scores most similar to the cluster averages were selected as titular breeds. Titular breeds for each cluster were the Maltese terrier, the Great Dane, the Akita, the Australian shepherd, the American Staffordshire terrier, and the Weimaraner. Using the AKC data, we tracked longitudinal trends in annual registration numbers of breeds of each cluster over the period from 1926 to 2005. This period was subdivided into periods with differing overall trends by fitting natural cubic splines to the overall raw trend and considering both the spline and its derivative curves. Differences in the absolute numbers of dogs and trends in registrations over nearly 80 years were identified: an Early period (1926–1944, during which total registration numbers were very low); a Mid-Century Period (1945–1971, during which total registration numbers were tending to rise from year to year); a First Decline (1972–1979, a brief period during which registration numbers experienced a trend of more gradual decline); a Recovery (1980–1992, where registration numbers began to gradually rise again); and a Second Decline (1993–2005, a second sustained period of falling registration numbers, more dramatic than the first decline). The current article describes the ways in which the clustered behaviour of dogs associate with these trends. That said, there is no compelling evidence that shifts in the popularity within or between the clusters reflect consumer canine behavioural preferences. Understanding historic trends in the demand for certain canine behavioural traits could help veterinary and urban animal management stakeholders to anticipate future needs for education and infrastructure.

## 1. Introduction

The predominant roles dogs play in human contexts have shifted from work to companionship [1]. Informal selection of favourable behavioural traits, such as herding, guarding, hunting, and attributes that suit companionship date back thousands of years. However, the proliferation of the more than 400 extant breeds can be traced to the mid-19th century when, fueled by the emergence of pedigree kennel clubs, the emphasis in dog breeding shifted from function to fashion [2]. Longitudinal changes in the annual numbers of purebred dogs registered with the American Kennel Club (AKC) offer a framework with which to examine the factors that affect our collective decisions about what breeds of dogs we want to live with. Like baby names, many shifts in breed popularity can be explained by a random drift model of cultural evolution [3]. Furthermore, as with other forms of popular culture, shifts in preferences for dogs conform to “the logic of fashion cycles” in that breeds that rapidly increase in popularity subsequently experience rapid decreases in popularity [4]. Changes in the popularity of different breeds are also influenced by the media, including hit movies featuring dogs and inadvertent promotions of certain breeds by royalty and celebrities [5,6].

Beliefs about breed-characteristic behavioural traits can also affect the popularity of breeds. For example, in a recent Danish study, owners of French Bulldogs and Cavalier King Charles Spaniels specifically indicated they were drawn to their pets because of these breeds’ reputations for being easy to live with [7]. However, when newly popular breeds are acquired by the uninitiated, the challenges of keeping dogs of breeds with strong behavioural traits may lead to amplified manifestation of unwelcome behaviours, premature euthanasia, and relinquishment to shelters, and the eventual decline in the popularity of those breeds. Indeed, the Danish study found that owners of Chihuahuas encountered more behaviour problems with their pets and, as a result, were more than twice as likely as French bulldog and Cavalier King Charles spaniel owners to say they would choose a different breed for their next dog.

Practical domestic matters are also likely to have influenced breed popularity over time. As humans have largely overcome the challenges of ectoparasite control, it is likely that dogs have been granted access to areas within homes that would otherwise have been denied them. At the same time, it may be that large, heavy-coated breeds that may be difficult to keep clean have gained access to the inside of homes more than occurred historically. It is even possible that the rise of efficient and increasingly inexpensive vacuum cleaners may have allowed the so-called shedding breeds to gain popularity. In a similar vein, the so-called non-shedding breeds are often loosely branded as hypoallergenic and so may have an appeal to those concerned about allergies, especially in children. A counter-point is that early exposure to companion animals may help lower rates of human allergies in later life [8].

Some dog breeds are arguably more likely to appeal to prospective owners who are driven by fashion. Their appearance may be more important than their behaviour, except perhaps for being sociable with unfamiliar humans and dogs, and biddable on the lead. The appeal of having a dog as an exercise companion has been emphasised [9] not least by those seeking to promote pet ownership, including pet-food manufacturers. Some dogs are better suited to this role than others. For example, brachycephalic breeds may be exercise-intolerant and thus unable to keep up with a jogging human. It is also possible that the role of dogs in leisure has been subject to longitudinal change with the rise of agility trials that, to some extent, may have challenged the popularity of obedience trials [10]. This shift may have promoted certain agile breeds over others. That said, both obedience trials and agility trials tend to favour dogs of the working breeds, such as kelpies and collies, probably because they have been selected for physical fitness and the ability to respond to humans while working at some distance from them. As human housing has become denser with urbanisation, it may be that smaller breeds have become more popular because of the perception that they require less space to meet their behavioural needs, even to the extent that they may be housed adequately in apartments with no outdoor area for toileting purposes. To minimise conflict with neighbours, the dogs in these contexts must be especially sociable with humans and conspecifics and have elevated thresholds for vocalisation.

The introduction of television and its transient rise and fall as the focal source of family entertainment may have made potential dog owners more aware of the variety of breeds available. By the same token, the media coverage of urban crime may have made some owners interested in owning breeds with reputations for protective aggression [11]. The rise of affable breeds, such as the Cavalier King Charles spaniel, known to be a low risk for aggression, may reflect publicity about the risks of canine aggression, or an increase in social pressure to avoid dog-bite incidents, and the increasing likelihood of litigation that may result from them. In addition, changes in occupational demands, family structure, and the rise of computer games may mean that people have less time than in the past for dog management and exercise. This may account for puppies being advertised online as being of breeds that allegedly do not need exercise (Claudia Jones, pers comm).

The current report shows how the demand for groups of breeds with various overarching physical and behavioural similarities have shifted over time in the United States. A particularly intriguing prospect is that, as the popularity of behavioural groups have changed, so too has the challenges of dog-keeping. These challenges reflect the common adaptive and maladaptive behavioural patterns experienced by pedigree dog owners, the owners of those crossbred dogs closely descended from the population, and the public who share public spaces with dogs.

## 2. Materials and Methods

### 2.1. Data

The American Kennel Club (AKC) provided one of us (HH) with the numbers of annual new registrations for 167 breeds of pedigree dogs between 1926 and 2005 (total *N* = 51,859,340 dogs). This data set has been used previously to investigate various aspects of shifts in breed popularity (See [12] for a summary).

We obtained behavioural data on 32,005 dogs through the University of Pennsylvania’s Canine Behavioural Assessment and Research Questionnaire (C-BARQ). C-BARQ is an international online 100-item survey that asks owners to describe their dogs’ behaviour and reactions to common events and stimuli using a series of 5-point ordinal rating scales. The current study was based on owner responses on 14 behavioural subscales (see Table 1) as well as 22 miscellaneous traits, behaviours, and responses (see Table 2).

### 2.2. Breed Selection for Cluster Analysis

The current cluster analysis was based on 103 breeds for which there were more than 50 individuals in the C-BARQ dataset. Of these breeds, 4 were excluded for being mixed breeds or unknown. These were those labelled as “mixed breed/unknown”, “pit bull mix”, “golden doodle” and “labradoodle”, and a further 13 were excluded for not matching any of the 150 or so breeds in the AKC data. These included dogs described by their owners as “American Pit Bull Terrier”, “Pit Bull”, “Rat Terrier”, “American Bulldog”, “Alaskan Husky”, “Australian Kelpie”, “Chinook”, “Spanish Water Dog”, “English Bulldog”, “English Shepherd”, “Dingo”, “Eurasier”, and “Treeing Walker Coonhound”.

Additional to these exclusions, several breeds were identified as special cases.

Greyhounds—Greyhounds were excluded because C-BARQ data were likely to include individuals from both racing and show lines, whereas AKC data included individuals primarily from show lines.

Schnauzers—In both the AKC and C-BARQ data, schnauzers were categorised as either giant, standard, or miniature. Giant schnauzers were excluded from the current study due to being represented in insufficient C-BARQ numbers (*n* = 31). In contrast, both miniature and standard schnauzers were included in the current final cluster analysis.

Collies—Rough, smooth, and tricolor variants are mentioned in the official breed standard. However, for registration purposes, they are categorised under the single “collie” category. The bearded and border collies were recognized only recently by the AKC (bearded collies in 1976 and border collies in 1995) and are not in the same breed category as “collies.” The AKC data in the current study are differentiated only between bearded collies, border collies, and collies, the last of which may reflect a grouping of those dogs categorised by C-BARQ as rough, collies and smooth collies. Only rough collies and collies had more than 50 C-BARQ entries, so smooth collies were excluded from the current analysis. Rough collies and collies grouped closely in a preliminary cluster analysis, and so both were included in the current final cluster analysis.

Dachshunds—While both “dachshunds” and “miniature dachshunds” had more than 50 C-BARQ records, the AKC data did not differentiate between these two varieties. However, dachshunds and miniature dachshunds grouped closely in a preliminary cluster analysis, so both were included in the current final cluster analysis.

Poodles—While “toy poodle”, “miniature poodle”, and “standard poodle” all had more than 50 C-BARQ entries, the AKC data does not differentiate between these three varieties. However, unlike collies and dachshunds, the three varieties of poodles did not group closely together in a preliminary cluster analysis. Therefore, all three were considered unsuitable for the current final cluster analysis, as there would be no way to attribute poodle registration numbers to any particular cluster. That said, poodles are included among “none” in the non-clustered charts below. The rise of poodles in the 1950s and 1960s represented the biggest boom in AKC history. While there are no statistics, it is believed that most of them were probably toys or miniatures.

After these exclusions, the remaining 82 of the 103 candidate breeds identified from C-BARQ were used in the final cluster analysis as represented breeds (See Table A1 in Appendix A).

### 2.3. Cluster Analysis

The cluster analyses were completed using R Statistical software [13].

Arithmetic means for each of the C-BARQ subscales and other miscellaneous behaviours (See Table 1 and Table 2) were calculated from the 50+ records for each of the 82 breeds.

A Euclidian distance matrix for these means was calculated using the dist() function of the ‘stats’ package. An agglomerative hierarchical cluster analysis was then performed on this matrix using the ()hclust function, also of the ‘stats’ package. Hierarchical clustering was selected in preference to flat clustering because it provides a more informative structure and does not require pre-specifying the number of clusters. A preliminary cluster analysis was performed specifically to see if breed variants such as collies and dachshunds (see above) clustered together, and once the included breeds were established from this preliminary analysis, the procedure was repeated.

**Figure 1:**
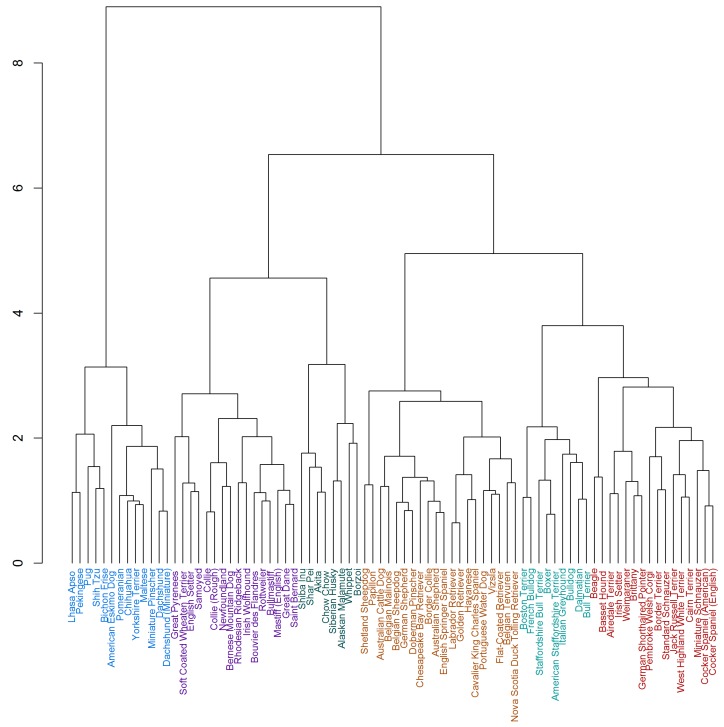
Dendrogram from Agglomerative Hierarchical Cluster analysis of C-BARQ behavioural subscales and miscellaneous behaviours for arithmetic means of 82 breeds. Division into six clusters is shown.

The resulting dendrogram may be viewed in Figure 1.

### 2.4. Selection of Titular Breeds and Cluster Clarification

The median heights of each behavioural cluster were determined from existing databases of breed standards. Height was selected as a morphological metric in preference to weight because modern trends in canine obesity [14] suggest that height is likely to have been more consistent than intrabreed weight over the period of 1926–2005. Where possible, height values were determined for each breed by averaging the values given on the AKC website (http://www.akc.org/dog-breeds/). When height values were not available from the AKC, they were gathered from other sources, (primarily, the Australian National Kennel Council (http://ankc.org.au/Breed/Index/).

Averages for each of the 14 behavioural subscales and 22 miscellaneous traits, behaviours, and responses were calculated for each cluster, and the breed with average scores most similar to the cluster average were selected as titular breeds. Titular breeds for the six clusters were the Maltese terrier, the Great Dane, the Akita, the Australian shepherd, the American Staffordshire terrier and the Weimaraner.

### 2.5. Trends in Clusters Over Time

The trends in AKC registration numbers of breeds of each cluster were tracked over the period of 1926–2005. This period was divided into periods with differing overall trends by fitting a natural cubic spline to the overall raw trend and considering this spline and its derivative curves.

## 3. Results

### 3.1. Size and Behavioural Features of Clusters

Average heights of the breeds in each cluster are shown in Table 3. Despite no direct size or weight variables being included among the traits upon which the cluster analysis was performed, dogs of similar size generally tended to cluster together (Anova F = 24.93, *p* < 0.001).

Behavioural cluster means for C-BARQ subscales (Table 4) and miscellaneous traits (Table 5) were also examined.

### 3.2. Trends in Clusters over Time

**Figure 2:**
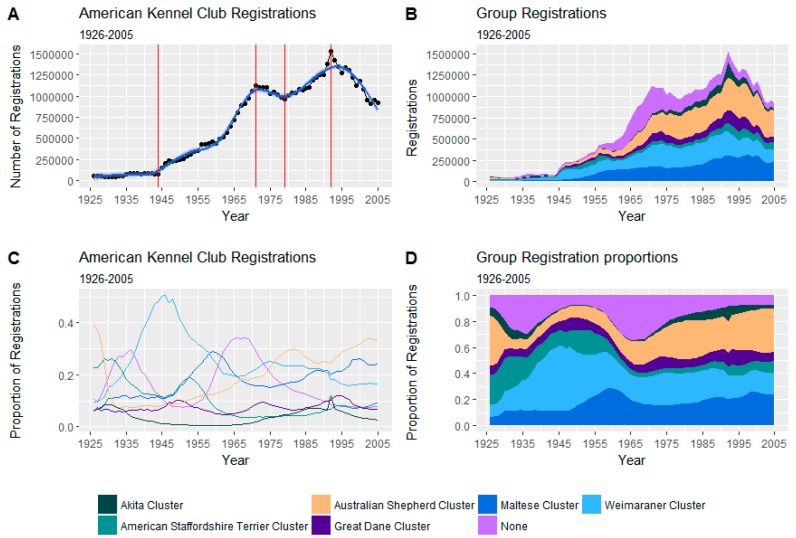
(**A**): Total American Kennel Club (AKC) registration numbers by year. Smaller windows (*n* = 5) with differing trends in overall registration numbers demarked by red lines. A fitted natural cubic spline (df = 9) is shown in blue. [The large increase in the “none” category between the 1950s and 1960s is largely due to the boom in the popularity of poodles. The subsequent growth in the Australian Shepherd cluster is chiefly due to slow but steady rise in Labrador and golden retrievers]; (**B**): Stacked Area plot of registrations grouped by cluster; (**C**): Annual proportion of registration by cluster; (**D**): Stacked Area plot of proportion of registration by cluster.

The total annual AKC registration numbers are shown in Figure 2.

#### 3.2.1. Maltese Cluster

The Maltese Cluster consists of 12 breeds, all small in stature, ranging from the Pomeranian at 15–18 cm (6–7 inches) to the American Eskimo Dog at up to 48 cm (19 inches) in its largest variety.

**Figure 3:**
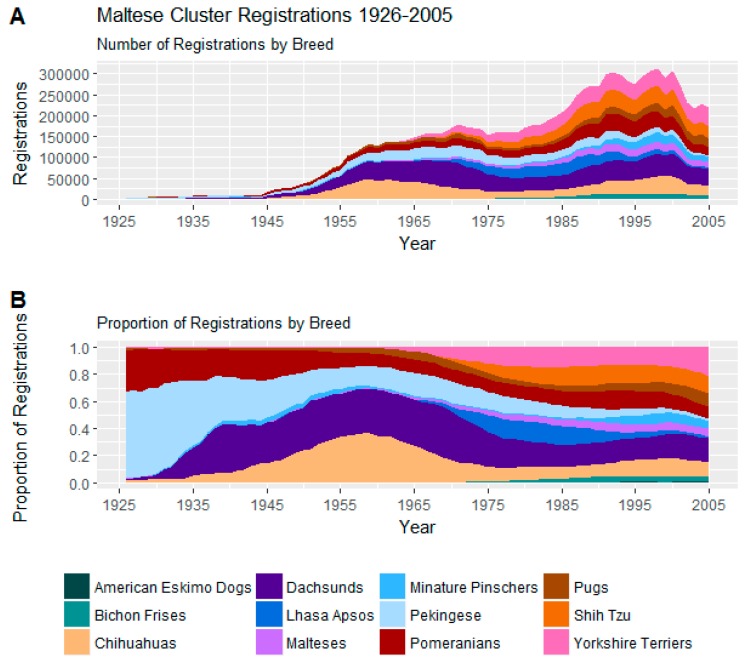
(**A**) Number of registrations; (**B**) and proportion of registrations within the Maltese Cluster.

Trends in registrations of these 12 breeds may be seen in Figure 3.

Early in the period of study, Pomeranians and Pekingese dominated registrations within this cluster, but, by the end of the study period, considerable diversity developed within this cluster. In 2005, Pomeranians and Pekingese represented only 8.8% and 1.6% of registrations in this cluster, respectively; down from 22.8% and 47.7% in 1935. Meanwhile, four other breeds had risen to represent over 10% of registrations each in this cluster by 2005. Dachshunds and then Chihuahuas began to rise in popularity, beginning around the mid-1930s, and later, around the late 1970s, Shih Tzu and Yorkshire terriers also rose in prominence. 

On the C-BARQ behavioural survey, dogs in this cluster score high on aggression-, fear-, and separation-based indices (See Table 4) compared to other clusters but are also relatively excitable, highly motivated by their owners’ attention, and likely to engage in social grooming.

Within the cluster, the Pekingese have high average scores for stranger-directed aggression (1.2), owner-directed aggression (0.7), dog-directed aggression (1.5), and dog rivalry (1.3) but tend to be somewhat less fearful than the Chihuahuas and dachshunds that began to dominate the group mid-centenary.

The Pomeranian, on the other hand, has average scores suggesting a more even temperament: stranger-directed aggression (0.8), owner-directed aggression (0.4), dog-directed aggression (1.0), and dog rivalry (0.7) and, on average, score high for trainability, but also lead the cluster in tendency to mark furniture with urine.

Cluster members subject to later increases in popularity, the Yorkshire terrier and particularly the Shih Tzu, show relatively low aggression and fear within the cluster.

#### 3.2.2. Weimaraner Cluster

The Weimaraner cluster is a group of 16 generally small-to-moderately sized breeds ranging from the Cairn terrier at 24–25.5 cm (9.5–10 inches) to the Irish Setter at 63.5–68.5 cm (25–27 inches).

**Figure 4:**
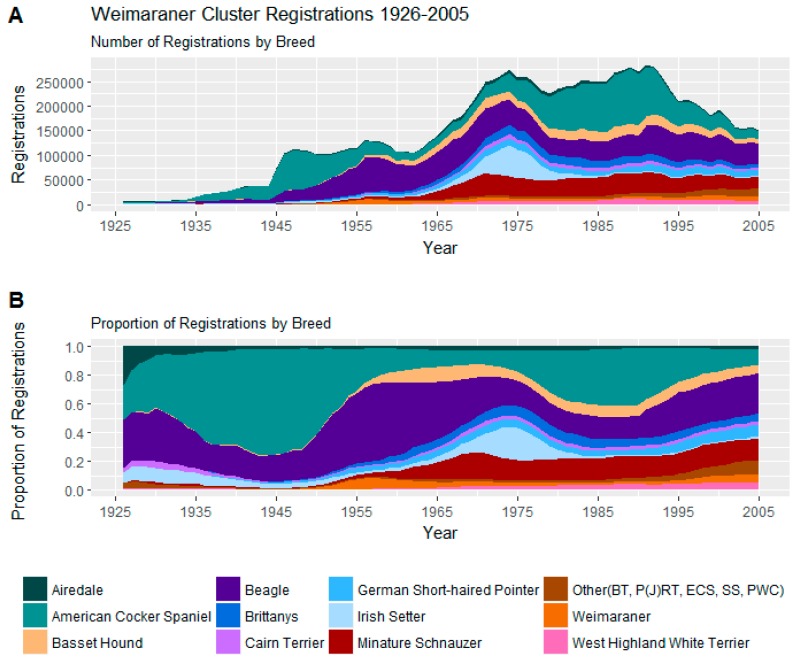
(**A**) Number of registrations; (**B**) and proportion of registrations within the Weimaraner Cluster. BT = Border Terrier, P(J)RT = Parson (Jack) Russell Terrier, SS = Standard Schnauzer, PWC = Pembroke Welsh Corgi.

Trends in registrations of these 16 breeds may be seen in Figure 4.

Registrations in the cluster were dominated by the perennially popular Beagle and also two surges in popularity of the American Cocker spaniel, the popularity of the Irish setter around the 1970s, and the ascendency of the miniature Schnauzer from the late 1960s onward.

On the C-BARQ behavioural survey, like the similarly statured American Staffordshire Cluster discussed below, the Weimaraner Cluster as a whole has moderate scores on the aggression and fear behavioural indices (See Table 4). What serves to distinguish the Weimaraner Cluster from the American Staffordshire Cluster is a pattern of propensity among the miscellaneous behaviours (see Table 5) which for this cluster has high average scores for the olfactory-related behaviours of food theft, rolling in faeces, and coprophagia.

Even within the cluster, Beagles score highly for these olfactory-related behaviours, averaging 1.9 for rolling in faeces, 1.3 for consuming faeces, and 1.8 for stealing food. They are also relatively aggressive towards owners and canine housemates compared with other Weimaraner Cluster breeds, but not towards unfamiliar dogs or humans. American Cocker spaniels and miniature schnauzers, on the other hand, score lower for olfactory-related behaviours, and both show more stranger-related aggression than beagles. The miniature schnauzers show less aggression towards their owners as do the relatively even-tempered, but food-theft prone, Irish Setters.

#### 3.2.3. American Staffordshire Terrier Cluster

The American Staffordshire Cluster contained 9 breeds of moderate size, ranging from the Italian greyhound at 33–38 cm (13–15 inches) to the Boxer at 54.5–63.5 cm (21.5–25 inches).

**Figure 5:**
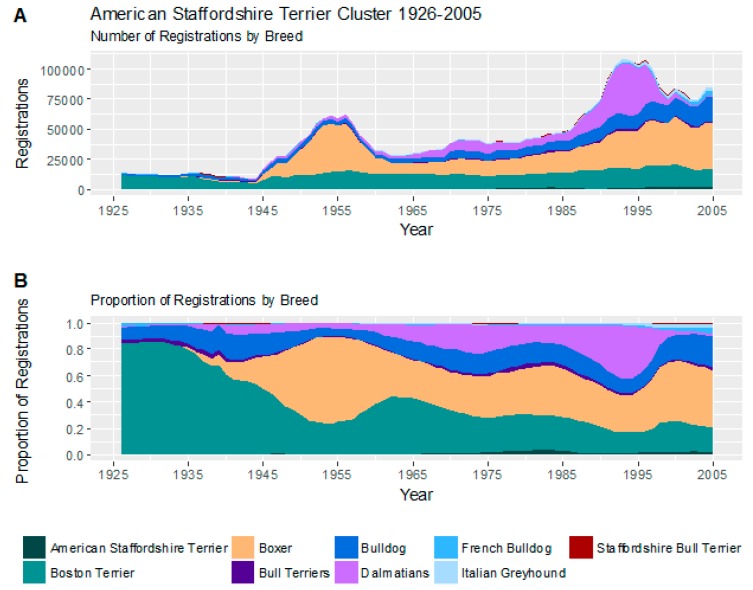
(**A**) Number of registrations; (**B**) and proportion of registrations within the American Staffordshire Cluster.

Trends in registrations of these 9 breeds may be seen in Figure 5.

Four breeds have dominated this cluster over time: the Boston terrier, the Boxer, the Bulldog and the Dalmatian. Interestingly, 3 of these 4 breeds are brachycephalic. The Boston terrier and, to a lesser degree, the Bulldog have been popular throughout the study period, whereas the Boxer experienced a significant surge in registrations in the post-war period, and after a slump, a gradual increase in popularity since around 1970. Dalmatians began to increase in popularity from the early 1960s, and experienced particular high registrations between the mid-1980s to mid-1990s. 

On the C-BARQ behavioural survey, this cluster is largely distinguished from the similarly statured Weimaraner Cluster by a penchant toward the visual-mediated stereotypies of staring and shadow-chasing, as well as a hyperactive temperament.

Within the cluster itself, the Boston terrier (and the less popular Italian greyhound) show high rates of indoor-marking, with averages more typical of the Maltese Cluster. However, the Boston terrier does share the relatively moderate aggression and fear indices of the cluster. The most aggressive breeds in the cluster are the Dalmatian and Boxer, while the most fearful are the Italian greyhound and the Bulldog.

#### 3.2.4. Great Dane Cluster

The Great Dane Cluster is a collection of 15 breeds, mostly large-to-giant in stature from the soft-coated wheaten terrier at 43–48 cm (17–19 inches) to the Irish wolfhound standing at least 76 cm (30 inches).

**Figure 6:**
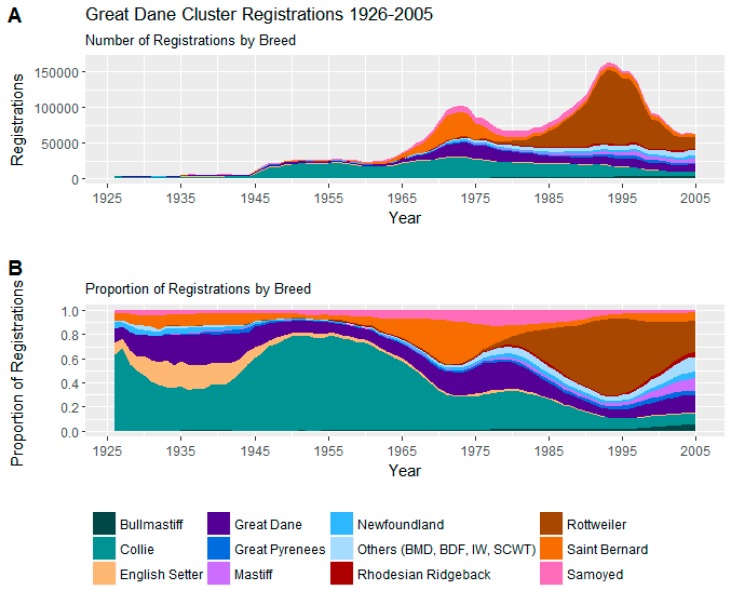
(**A**) Number of registrations; (**B**) and proportion of registrations within the Great Dane Cluster.

Trends in registrations of these 15 breeds may be seen in Figure 6.

Prominent breeds from this cluster include the modestly popular collie, the Saint Bernard which experienced relative popularity from the mid-1960s to the late 1970s, leading to the popularity of the Rottweiler from the 1980s forward.

On average, this cluster shows very low scores on aggression- and fear-based indices and low tendencies towards other problematic behaviours, and exhibits relatively trainable temperaments.

Within the cluster, the Rottweiler scores high averages for aggression indices and the Saint Bernard scores high averages for fear. The popular Collie and Rottweilers score highly for trainability, whereas the Saint Bernard has a low trainability score for this cluster. The less popular Newfoundland was characterised by low scores for aggression and fear within the cluster.

#### 3.2.5. Akita Cluster

The smallest cluster, the Akita Cluster, included 8 mostly large breeds of dog from the Shiba Inu at 34–42 cm (13.5–16.5 inches) to the Borzoi which stands from 66 cm (26 inches).

**Figure 7:**
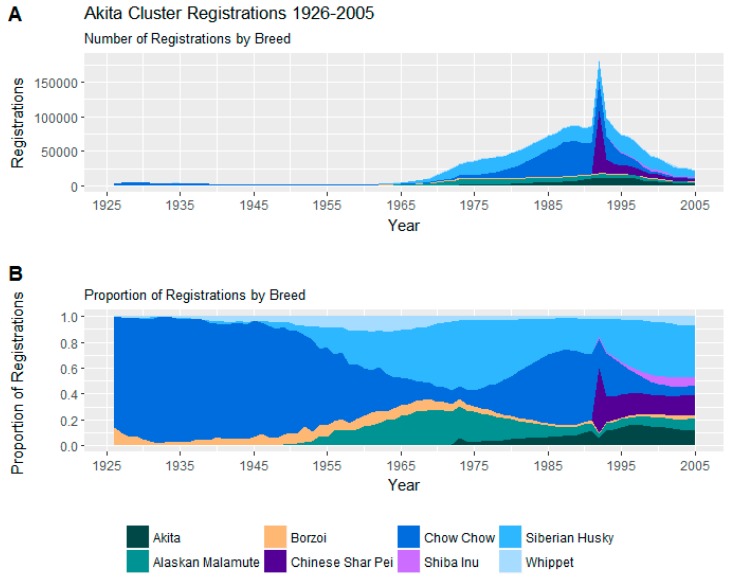
(**A**) Number of registrations; (**B**) and proportion of registrations within the Akita Cluster. The spike around 1992 reflects the rise of the Shiba Inus and Chinese Shar-Peis. When a new breed is first recognized by the AKC, there is an immediate spike in registrations because adult dogs are registered as well as new puppies. Because this cluster is relatively small, the spike is proportionately obvious.

Trends in registrations of these 8 breeds may be seen in Figure 7.

The Chow Chow and Siberian husky were prominent breeds in the cluster over the study period. On average, the cluster scores moderately on aggression and fear indices, appearing somewhat prone to owner and dog aggression and less amenable to training than the similarly statured Australian Shepherd Cluster discussed below. They are also low on attachment and attention-seeking and appear particularly prone to chasing, both their own tail and smaller mammals, and escaping from their homes and yards.

Within the cluster, the Siberian husky scores relatively low for aggression and fear compared with the Chow Chow, but is more prone to roam. The Borzoi scores low for aggression and fear, but is relatively difficult to train, and the Whippet is trainable and relatively non-aggressive, but somewhat prone to fear. 

#### 3.2.6. Australian Shepherd Cluster

The largest cluster, the Australian Shepherd Cluster, is composed of 20 mostly larger breeds but ranging from the Papillon 20.5–28 cm (8–11 inches) to the Doberman Pinscher 61–71 cm (24–28 inches).

**Figure 8:**
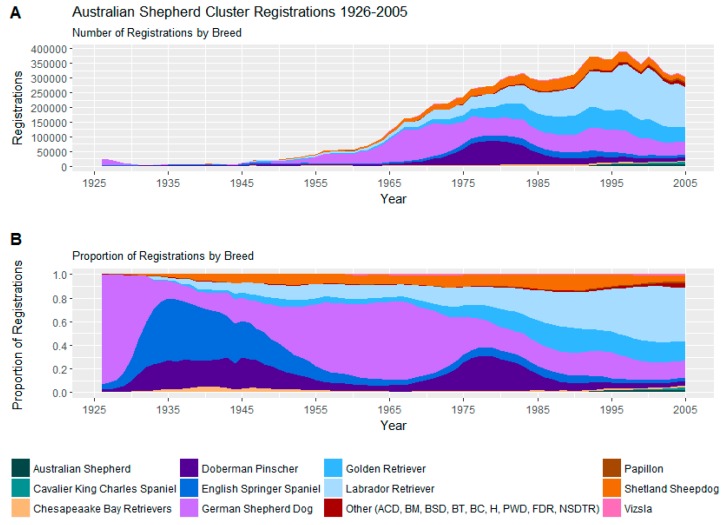
(**A**) Number of registrations; (**B**) and proportion of registrations within the Australian Shepherd cluster.

Trends in registrations of these 20 breeds may be seen in Figure 8.

This cluster contains some of the most currently popular breeds in the USA including the Labrador retriever, the German shepherd dog and the Golden retriever [15]. Notwithstanding a sharp decline in popularity in the pre-war years, the German shepherd dog has been a prominent breed throughout the survey period of 1926–2005, and the Labrador and Golden retrievers have been growing in prominence since the 1970s.

Behaviourally, this cluster is, on average, energetic, very trainable and has relatively low aggression towards their owners and other dogs, although somewhat more aggression towards strangers, and a low propensity towards many of the miscellaneous behaviours studied, apart from barking, for which it scores moderately.

Within the cluster, low aggression breeds include the Labrador Retriever, Golden Retriever, Portuguese Water Dog, Cavalier King Charles Spaniel and the Nova Scotia Duck Tolling Retriever. Breeds with high aggression generally included the Australian Cattle Dog, the Belgian Malinois and the Chesapeake Bay retriever, while the German Shepherd Dog showed high dog aggression and the Papillon high owner aggression. High fear breeds within the cluster included the Border Collie, Papillon and Shetland Sheepdog. The smaller Papillon and Shetland Sheepdog were also more prone to barking than the larger Golden Retriever and Labrador Retriever.

#### 3.2.7. Unclustered Breeds

**Figure 9:**
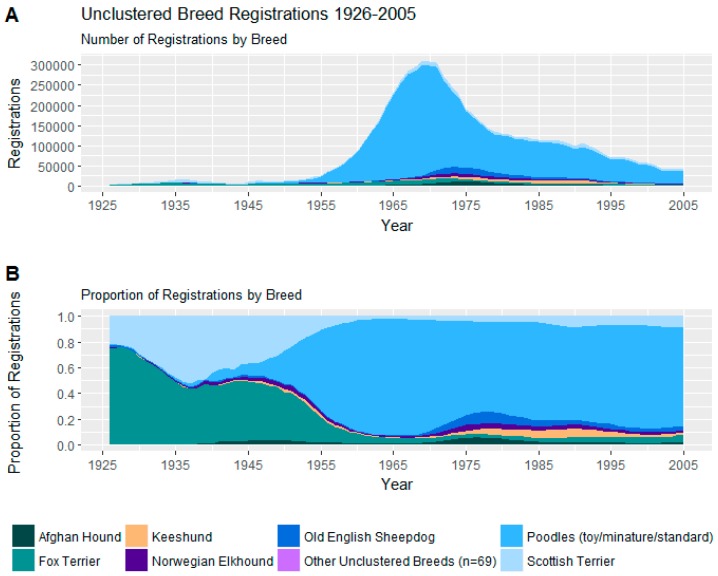
(**A**) Number of registrations; (**B**) and proportion of registrations within the unclustered breeds.

While most popular breeds over the study period were included in the cluster analysis, several popular breeds could not be included. Trends in registrations of these breeds may be seen in Figure 9. Particularly of note are the Poodles, which were very prominent mid-to-late century and the Fox Terrier and Scottish Terrier which were popular early breeds.

### 3.3. Behavioural Clusters of the Historically Most Popular Breeds

**Figure 10:**
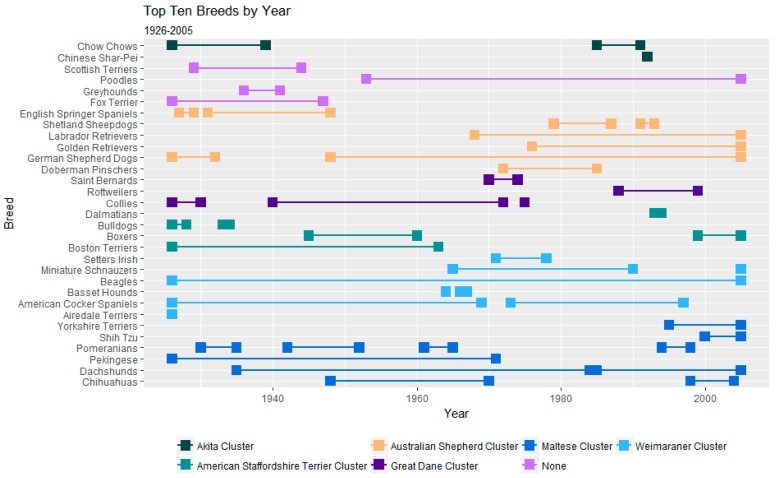
Top Ten Breeds by registration number every year from 1926 to 2005.

As shown in Figure 10, from 1926 to 2005, 31 breeds appeared in the top ten in terms of absolute registration numbers per year. There are representatives from all behavioural clusters among these 31 breeds, although only the Maltese cluster, the Weimaraner cluster and the Australian Shepherd cluster have a representative in every year’s top ten registrations.

## 4. Discussion

The current study has identified differences in the absolute numbers of dogs, and trends in rates of breed registrations, over a span of nearly 8 decades between 1926 and 2005. Within this overall span, various subperiods can be discerned: an Early period (1926–1944, during which total registration numbers were very low); a Mid-Century Period (1945–1971, during which total registration numbers were tending to rise from year to year); a First Decline (1972–1979, a brief period during which registration numbers experienced a more gradual decline); a Recovery (1980–1992, where registration numbers began to gradually rise again); and a Second Decline (1993–2005, a second sustained period of falling registration numbers, more dramatic than the first decline).

The large Australian Shepherd Cluster has been the most popular cluster since the 1970s. Between 1972 and 1979, when total registration numbers fell 12% from 1,101,943 to 965,250, Australian Shepherd Cluster registrations grew 29% from 213,348 to 275,266 and the cluster overtook the Weimaraner Cluster as the most popular cluster. The Irish setters and German short-haired pointers were prominent in the Weimaraner Cluster at the time but began to decline in popularity. Similarly, the low-aggressive, but more trainable and less energetic and escape-prone Labrador retriever and golden retrievers began to rise in popularity to positions they retained to the end of the study period and beyond. Even during the sharp registration decline of 1993–2005, where annual registrations fell 35% from 1,422,559 to 921,129, the Australian Shepherd Cluster underwent only a 19% decline from 375,804 to 304,271, the least of any cluster. It should be emphasised that the popularity of this group was primarily driven by Labrador and golden retrievers.

The Maltese Cluster finished the study period as the second most popular cluster, having overtaken the Weimaraner Cluster in 1991, and spent the most of the study period among the top three clusters. The cluster was particularly prominent around 1960, briefly becoming the most popular cluster as registrations of breeds in the Weimaraner Cluster (particularly of the previously ascendant American Cocker Spaniel) fell or stagnated, while the dachshund continued to grow in popularity and Chihuahuas rose quite quickly to prominence. Unlike the Australian Shepherd Cluster, the Maltese Cluster did not continue to grow during the First Decline period, falling about 8% from 172,669 to 159,556, and its second decline a relatively low 26% compared to the 35% of all registrations.

After peaking in the late 1940s, the Weimaraner Cluster finished the study period as the third most popular cluster. This early popularity, mostly because of the American Cocker Spaniel registrations, began to falter around the mid-century as breeds in the Australian Shepherd Cluster (especially the German Shepherd Dog) and the Maltese Cluster rose to prominence. In other words, as the moderately tempered, moderately sized American Cocker Spaniel fell in prominence, both smaller breeds and the larger, more trainable, marginally more even tempered German shepherd dog began to rise. At about the same time, within the Weimaraner Cluster itself, the beagle was rising in popularity to levels it would retain until the end of the study period and beyond. In the 1960s, Weimaraner cluster registrations began to rise again, this time in the form of the Irish setters and German shorthaired pointers, which would themselves fall as the retrievers began to rise in the 1970s.

The American Staffordshire Cluster finished the study just ahead of the Great Dane Cluster in fourth place. Over time, this cluster has suffered a gradual fall in prominence from the beginning of the study to about the mid-1970s, mitigated a little by the popularity of Boxers in the 1950s, although the Boston terrier has largely maintained its modest, yet steady popularity throughout the study period. There is little evidence of the First Decline in this cluster with registration numbers being more or less stagnant across this period. From the late 1970s onwards, boxers and bulldogs as important breeds within this cluster, experienced modest growth, and Dalmatians underwent a surge of popularity in 1990s. The effect of the second decline is modest at about 21%.

The prominence of the Great Dane Cluster over time has been relatively steady, consisting of between 4.7 and 12.0% of registrations throughout the study period. While this cluster includes many very large gentle dogs, popularity over time within this cluster has shifted from the relatively gentle collie towards the Rottweiler which, despite clustering in the Great Dane Cluster, has aggression and trainability averages similar to many of the Australian Shepherd Cluster breeds, and the Rottweiler is reported as generally more aggressive (and trainable) than the extremely popular Labrador and golden retrievers. It is also interesting that the Second Decline struck strongly in this cluster, registrations dropping 61% from 164,684 to 62,727, and Rottweiler registrations accounted for much of this drop (a drop of 88,244 registrations out of the 101,957 drop). That said, other breeds, such as mastiffs and Bernese mountain dogs, grew in registration during this decline period.

The Akita Cluster consisting of only a small number of breeds was the least prominent cluster at the conclusion of the study period and, indeed, had been for much of the study duration. This cluster’s registrations peaked around 1990, preceded by a gradual increase and a followed by a somewhat gradual decrease. Part of its unusual shape is explained by the AKC not recognising the Chinese Shar-Pei until 1992 [16].

This report describes the manner in which the trends in breed preferences over the years 1926–2005 manifested when considered at the level of the behavioural cluster. There is no compelling evidence that shifts in the popularity within or between clusters are caused by consumer preferences in animal behaviour. However, when such consumer preferences arise they must have some impact on the demand for different breeds, and thus alter the behavioural profile of the general pedigree canine population accordingly. Understanding how historic trends in how demand, whatever its causes, has affected the prevalence of certain canine behavioural traits could help veterinary and urban animal management stakeholders anticipate needs for education and infrastructure.

It is worth emphasizing that a single breed could become very popular because of random chance or a movie rather than on the basis of preferences for behavioural traits or physical attributes. However, in the present analysis, it might appear that people are selecting a group of breeds based on shared behavioral traits, whereas in reality they are actually opting, en masse, for a single breed because of a fad (e.g., the current boom in the popularity of French bulldogs) or perhaps a movie. 

Also, we note how hard it is to disentangle size from behavioural traits. Small dogs could become popular either because of fads for a few small breeds or because people are making rational decisions based on changing lifestyles.

“A potential limitation of the current work is that, in order to improve the accuracy of average owner scores, it used international owner responses for breed behaviour, while looking at trend data from only the AKC. The authors believe that the potential for any international discrepancy in either dog behaviour or owner ratings was mitigated by the breadth of the international data. Comparing international trends in canine behaviour may be a worthwhile future application of the C-BARQ project.”

Social scientists may integrate the trends and periods reported here with data on disposable income, leisure time, stay-at-home parenting and/or outdoor activity, in order to explore what factors may be influencing breed preferences and specifically behavioural preferences. Monitoring demand for certain canine behavioural traits could help veterinary and urban animal management stakeholders plan for anticipated needs in terms of equipment, education, and infrastructure. Assuming stability in the value placed on canine behavioural traits over time may represent a flaw. It would be prudent to explore preferences, both behavioural and otherwise, among the public at regular intervals, and ensure that upcoming veterinary and community infrastructure and resources are planned with the behavioural consequences of these preferences in mind. 

## Figures and Tables

**Table 1:** Canine Behavioural Assessment and Research Questionnaire (C-BARQ) Behavioural Subscales.

C-BARQ Name	Full Name	Description
Train	*Trainability*	Dog shows a willingness to attend to the owner and obey simple commands. Dog is not easily distracted, tends to be a fast learner, responds positively to correction, and will fetch or retrieve objects.
StrDirAgg	*Stranger-directed aggression*	Dog shows threatening or aggressive responses to strangers approaching or invading the dog’s or the owner’s personal space, territory, or home range.
OwnDirAgg	*Owner-directed aggression*	Dog shows threatening or aggressive responses to the owner or other members of the household when challenged, manhandled, stared at, stepped over, or when approached while in possession of food or objects.
DogDirAgg	*Dog-directed aggression*	Dog shows threatening or aggressive responses when approached directly by unfamiliar dogs.
FamDogAgg	*Dog rivalry*:	Dog shows aggressive or threatening responses to other familiar dogs in the same household.
DogDirFear	*Dog-directed fear*	Dog shows fearful or wary responses when approached directly by unfamiliar dogs.
StrDirFear	*Stranger-directed fear*:	Dog shows fearful or wary responses when approached directly by strangers.
NonSocFear	*Non-social fear*	Dog shows fearful or wary responses to sudden or loud noises (e.g., thunder), traffic, and unfamiliar objects and situations.
TouchSen	*Touch sensitivity*	Dog shows fearful or wary responses to potentially painful or uncomfortable procedures, including bathing, grooming, nail-clipping, and veterinary examinations.
SepRelProb	*Separation-related behaviour*	Dog vocalizes and/or is destructive when separated from the owner, often accompanied or preceded by behavioural and autonomic signs of anxiety including restlessness, loss of appetite, trembling, and excessive salivation
Excite	*Excitability*:	Dog displays strong reaction to potentially exciting or arousing events, such as going for walks or car trips, doorbells, arrival of visitors, and the owner arriving home; has difficulty calming down after such events.
AtcAtnSeek	*Attachment and attention-seeking*:	Dog maintains close proximity to the owner or other members of the household, solicits affection or attention, and displays agitation when the owner gives attention to third parties.
Chasing	*Chasing*	Dog chases cats, birds, and/or other small animals, given the opportunity.
Energy	*Energy level*	Dog is energetic, “always on the go”, and/or playful.

**Table 2:** Canine Behavioural Assessment and Research Questionnaire (C-BARQ) miscellaneous behaviours, reactions and traits.

C-BARQ Name	Description
EscapeRoam_77	Escapes or would escape from home or yard given the chance.
Rolling_78	Rolls in animal droppings or other ‘smelly’ substances.
Coprophagia_79	Eats own or other animals’ droppings or feces.
Chewing_80	Chews inappropriate objects.
Mounting_81	Mounts’ objects, furniture, or people.
Begging_82	Begs persistently for food when people are eating.
FoodSteal_83	Steals food.
FearStairs_84	Nervous or frightened on stairs.
PullLeash_85	Pulls excessively hard when on the leash.
MarkUrine_86	Urinates against objects/furnishings in your home.
SubEmoUrn_87	Urinates when approached, petted, handled, or picked up.
SepUrn_88	Urinates when left alone at night, or during the daytime.
SepDef_89	Defecates when left alone at night, or during the daytime.
Hyper_90	Hyperactive, restless, has trouble settling down.
Staring_93	Stares intently at nothing visible.
SnapFlies_94	Snaps at (invisible) flies.
TailChase_95	Chases own tail/hind end.
ShadowChase_96	Chases/follows shadows, light spots, etc.
Barking_97	Barks persistently when alarmed or excited.
GroomSelf_98	Licks him/herself excessively.
GroomOthers_99	Licks people or objects excessively.
OtherStereotypic_100	Displays other bizarre, strange, or repetitive behaviour(s)

**Table 3:** Cluster median, minimum, and maximum heights (ordered from left to right by median cluster height).

Cluster	Maltese	Weimaraner	American Staffordshire Terrier	Akita	Australian Shepherd	Great Dane
***n* of breeds**	13	16	9	8	20	16
**Median breed average (cm)**	22.86	36.85	38.10	53.02	54.61	64.77
**Minimum (cm) breed average**	13.97	24.77	30.48	38.10	24.13	45.72
**Maximum (cm) breed average**	35.56	66.04	59.06	68.58	66.04	78.74

**Table 4:** Cluster average C-BARQ scores for behavioural subscales (ordered from left to right by median cluster height).

Cluster	Maltese	Weimaraner	American Staffordshire Terrier	Akita	Australian Shepherd	Great Dane
**Train**	2.28	2.49	2.52	2.35	2.88	2.60
**StrDirAgg**	0.95	0.59	0.50	0.49	0.58	0.49
**OwnDirAgg**	0.36	0.20	0.18	0.20	0.13	0.10
**DogDirAgg**	1.21	0.99	1.11	1.12	0.92	0.78
**FamDogAgg**	0.76	0.62	0.62	0.66	0.55	0.43
**DogDirFear**	1.10	0.78	0.85	0.60	0.74	0.55
**StrDirFear**	0.97	0.54	0.61	0.60	0.58	0.47
**NonSocFear**	0.98	0.78	0.83	0.69	0.69	0.67
**TouchSen**	1.02	0.74	0.69	0.79	0.61	0.51
**SepRelProb**	0.81	0.64	0.62	0.50	0.49	0.43
**Excite**	2.27	2.16	2.09	1.78	2.09	1.81
**AtcAtnSeek**	2.22	1.97	2.06	1.64	2.07	1.81
**Chasing**	1.87	2.42	2.10	2.61	2.06	1.86
**Energy**	1.89	2.10	2.13	1.66	2.22	1.69
**EscapeRoam_77**	1.59	1.73	1.33	1.96	1.02	1.20

**Table 5:** Cluster average C-BARQ scores for miscellaneous behaviours (ordered from left to right by median cluster height).

Cluster	Maltese	Weimaraner	American Staffordshire Terrier	Akita	Australian Shepherd	Great Dane
**Rolling_78**	1.16	1.49	0.88	1.01	1.13	0.71
**Coprophagia_79**	0.87	0.93	0.89	0.77	0.91	0.68
**Chewing_80**	0.92	0.99	1.12	0.85	0.85	0.81
**Mounting_81**	0.67	0.37	0.50	0.28	0.34	0.22
**Begging_82**	1.76	1.52	1.57	1.21	1.28	1.14
**FoodSteal_83**	1.09	1.19	0.96	0.92	0.96	0.82
**FearStairs_84**	0.60	0.31	0.37	0.34	0.31	0.47
**PullLeash_85**	1.39	1.59	1.50	1.39	1.24	1.16
**MarkUrine_86**	0.66	0.30	0.37	0.22	0.17	0.14
**SubEmoUrn_87**	0.34	0.19	0.18	0.12	0.14	0.07
**SepUrn_88**	0.82	0.40	0.52	0.25	0.21	0.19
**SepDef_89**	0.67	0.28	0.40	0.23	0.17	0.16
**Hyper_90**	0.85	0.76	0.92	0.53	0.80	0.48
**Staring_93**	0.65	0.55	0.66	0.49	0.39	0.35
**SnapFlies_94**	0.31	0.29	0.26	0.24	0.19	0.15
**TailChase_95**	0.48	0.25	0.39	0.51	0.31	0.27
**ShadowChase_96**	0.44	0.37	0.48	0.35	0.36	0.28
**Barking_97**	2.00	1.57	1.11	0.79	1.44	1.13
**GroomSelf_98**	1.13	0.75	0.93	0.62	0.57	0.57
**GroomOthers_99**	1.18	0.63	0.96	0.49	0.62	0.39
**OtherStereotypic_100**	0.56	0.40	0.48	0.31	0.36	0.23

## Appendix A

**Table A1:** Number of Canine Behavioural Assessment and Research Questionnaire (C-BARQ) Records for the breeds included in the cluster analysis.

Breed	C-BARQ Responses
Airedale Terrier	142
Akita	210
Alaskan Malamute	104
American Eskimo Dog	69
American Staffordshire Terrier	173
Australian Cattle Dog	372
Australian Shepherd	641
Basset Hound	104
Beagle	318
Belgian Malinois	173
Belgian Sheepdog	58
Belgian Tervuren	129
Bernese Mountain Dog	210
Bichon Frise	180
Border Collie	829
Border Terrier	88
Borzoi	53
Boston Terrier	126
Bouvier des Flandres	55
Boxer	345
Brittany	137
Bull Terrier	74
Bulldog	74
Bullmastiff	103
Cairn Terrier	98
Cavalier King Charles Spaniel	144
Chesapeake Bay Retriever	51
Chihuahua	477
Chow	88
Cocker Spaniel (American)	275
Cocker Spaniel (English)	210
Collie	233
Collie (Rough)	94
Dachshund	235
Dachshund (Miniature)	161
Dalmatian	120
Doberman Pinscher	441
English Setter	88
English Springer Spaniel	220
Flat-Coated Retriever	79
French Bulldog	92
German Shepherd	1299
German Shorthaired Pointer	114
Golden Retriever	974
Great Dane	214
Great Pyrenees	94
Havanese	160
Irish Setter	71
Irish Wolfhound	55
Italian Greyhound	54
Jack Russell Terrier	370
Labrador Retriever	1835
Lhasa Apso	80
Maltese	176
Mastiff (English)	218
Miniature Pinscher	133
Miniature Schnauzer	224
Newfoundland	363
Nova Scotia Duck Tolling Retriever	78
Papillon	122
Pekingese	51
Pembroke Welsh Corgi	159
Pomeranian	213
Portuguese Water Dog	141
Pug	182
Rhodesian Ridgeback	176
Rottweiler	566
Saint Bernard	67
Samoyed	52
Shar Pei	51
Shetland Sheepdog	344
Shiba Inu	260
Shih Tzu	265
Siberian Husky	283
Soft Coated Wheaten Terrier	325
Staffordshire Bull Terrier	254
Standard Schnauzer	59
Vizsla	103
Weimaraner	133
West Highland White Terrier	112
Whippet	184
Yorkshire Terrier	225
